# Analyses of* Physcomitrella patens* Ankyrin Repeat Proteins by Computational Approach

**DOI:** 10.1155/2016/9156735

**Published:** 2016-06-27

**Authors:** Niaz Mahmood, Nahid Tamanna

**Affiliations:** ^1^Graduate Program in Experimental Medicine, McGill University, Montreal, QC, Canada H2X 0A8; ^2^Graduate Program in Biological Sciences, University of Manitoba, Winnipeg, MB, Canada R3T 2N2

## Abstract

Ankyrin (ANK) repeat containing proteins are evolutionary conserved and have functions in crucial cellular processes like cell cycle regulation and signal transduction. In this study, through an entirely in silico approach using the first release of the moss genome annotation, we found that at least 54 ANK proteins are present in* P. patens*. Based on their differential domain composition, the identified ANK proteins were classified into nine subfamilies. Comparative analysis of the different subfamilies of ANK proteins revealed that* P. patens* contains almost all the known subgroups of ANK proteins found in the other angiosperm species except for the ones having the TPR domain. Phylogenetic analysis using full length protein sequences supported the subfamily classification where the members of the same subfamily almost always clustered together. Synonymous divergence (dS) and nonsynonymous divergence (dN) ratios showed positive selection for the ANK genes of* P. patens* which probably helped them to attain significant functional diversity during the course of evolution. Taken together, the data provided here can provide useful insights for future functional studies of the proteins from this superfamily as well as comparative studies of ANK proteins.

## 1. Introduction

Ankyrin (ANK) repeats, composed of around 30–34 amino acids, are evolutionary conserved protein domains found to be involved in mediating protein-protein interactions [1]. In metazoans, the ANK repeat containing proteins has diversified functions in important processes like signal transduction, cell-cycle regulation, maintaining the integrity of cytoskeleton, transcriptional regulation, inflammatory response, development, and different types of cellular transport mechanisms [2]. Defect in ANK proteins has been found in a number of human diseases. For example, the ankyrin repeat domain 11 (ANKRD11) proteins interact with and also enhance the transcriptional activity of p53. In breast cancer cell lines, the expression level of ANKRD11 decreases compared to controls [3]. Ankyrin dysfunction has been linked with fatal human arrhythmias, such as the “ankyrin-B syndrome” in which there is an aberration of the human ankyrin-B gene (ANK2) [4].

The importance of ANK repeats can be underlined by their abundance in virtually all phyla. In photosynthetic organisms, these proteins have also been shown to be involved in a number of important physiological processes. Zhang and colleagues first reported on a light-dependent plant ANK protein which is involved in cell differentiation and development in* Arabidopsis* [5]. EMB506, a five-ANK repeat containing protein, has been shown to be essential for embryogenesis in* Arabidopsis* [6]. Another ANK protein, known as BOP1, is required for leaf morphogenesis [7]. XBAT32 and XBAT35 are linked with the regulation of ethylene biosynthesis [8, 9] and ethylene signaling [10], respectively. Several ANK proteins have been demonstrated to play role in responses to biotic and abiotic stresses in plants. The expression of rice* OsBIANK1* gene, encoding proteins containing ANK repeats, is altered in pathogen infected rice-seedlings compared to that of the controls which suggests its involvement in disease resistance response [11]. Furthermore, Yan and colleagues have shown that the* Arabidopsis* ANK protein, AKR2, might be involved in regulating antioxidant metabolism during disease resistance and stress responses [12].

The recent advancement in genome sequencing has enabled the genome-wide identification and characterization of ANK proteins from several photosynthetic species like* Arabidopsis* [14], rice [15], and tomato [16]. The availability of the genome sequence of* Physcomitrella patens* [17] provided us with an excellent opportunity for a genome-wide analysis of this ANK family in bryophyte. Here, we report analyses of the ANK proteins of* P. patens* using first release of the moss genome annotation.

## 2. Methods

### 2.1. Data Retrieval and Identification of ANK Proteins

The publicly available protein sequences of* P. patens* were downloaded from the JGI Phytozome database (first release of the moss genome annotation) [18] and domain annotation of these proteins was done by InterProScan [19]. Then* ANK* proteins were screened by searching for the PF00023 domain using an in-house Perl script as described in a previous paper [20]. BLASTP was carried out with NCBI* nonredundant protein database* using the sequences retrieved from InterProScan as queries. After that, the candidate sequences were curated manually using available annotations in GenBank and existing literature. The molecular weights and isoelectric points were determined separately from online web server (http://www.bioinformatics.org/sms2/). Subcellular localization was predicted by the online web server of ProtComp 9.0 (http://www.softberry.com/berry.phtml?topic=protcomppl&group=help&subgroup=proloc).

### 2.2. Classification and Phylogenetic Analyses of the ANK Proteins

The proteins were classified into different subgroups based on the presence of additional conserved domains other than the ANK domain as described previously [15, 21]. Phylogenetic tree file was constructed by the online webserver, SATCHMO-JS [22]; and the tree was visualized by the Molecular Evolutionary Genetics Analysis (MEGA) software version 4.1 [23]. In addition, synonymous and nonsynonymous substitution pattern were determined as described previously [24].

## 3. Results and Discussion

**Figure 1:**
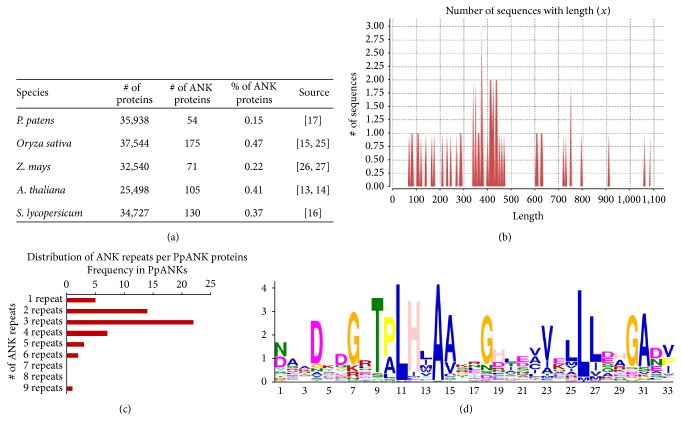
(a) The total number of predicted ANK proteins identified by different groups in the four sequenced angiosperm genomes (*Oryza sativa, Zea mays, Arabidopsis thaliana,* and* Solanum lycopersicum*) along with their bryophyte counterpart* P*.* patens*. The total number of predicted proteins of each species is also provided. See source column for references. (b) Distribution of PpANK proteins according to their length. (c) Number of putative ANK repeats per protein shown graphically by bar diagram. The horizontal axis in the figure represents different numbers of ANK repeats while the vertical axis represents the frequency of the proteins corresponding to different number of repeats. A large percentage (41%) of the PpANKs have 3 repeats within their sequence, as seen in the graph. (d) The consensus sequence of the* P. patens* ankyrin repeat motif.

Using our approach, we were able to identify a total of 54 proteins having at least one ANK repeat in* P. patens* (in the first released annotation of the moss genome). The identified sequences were further verified in a reiterative process through manual curation. The percentage of ANK proteins in* P. patens* (0.15%) is a bit lower compared to the other species from the tracheophyte lineage as listed in Figure 1(a).

The identified sequences from* P. patens* were designated as PpANK1, PpANK2,…,PpANK54, respectively, for analysis purpose during this study (Table 1).  Figure 1(b) shows the distribution of the PpANKs according to the number of amino acids they contain within their primary sequence. The largest protein (PpANK8) had a length of 1,088 amino acids, while the shortest one (PpANK48) contained only 74 amino acids. The molecular weights (MW) and isoelectric points (PI) of the PpANK proteins deduced from their protein sequences are listed in Table 1. In addition, it was observed that these 54 PpANK proteins contained a total of 163 ANK repeats among themselves. The number of ANK repeats per protein in* P. patens* ranged in between 1 and 9, whereas the average number of repeats per protein was 3. The frequency of the proteins having different number of ANK repeats is shown in Figure 1(c). The highest number of repeats (9) was found in PpANK43 whereas PpANK4, PpANK18, PpANK22, PpANK33, and PpANK49 had just one ANK repeat motif each. In general, most ANK proteins have two to six repeats; and the largest known number of repeats is 34 that was found in a* Giardia lamblia* protein [25].

**Figure 2:**
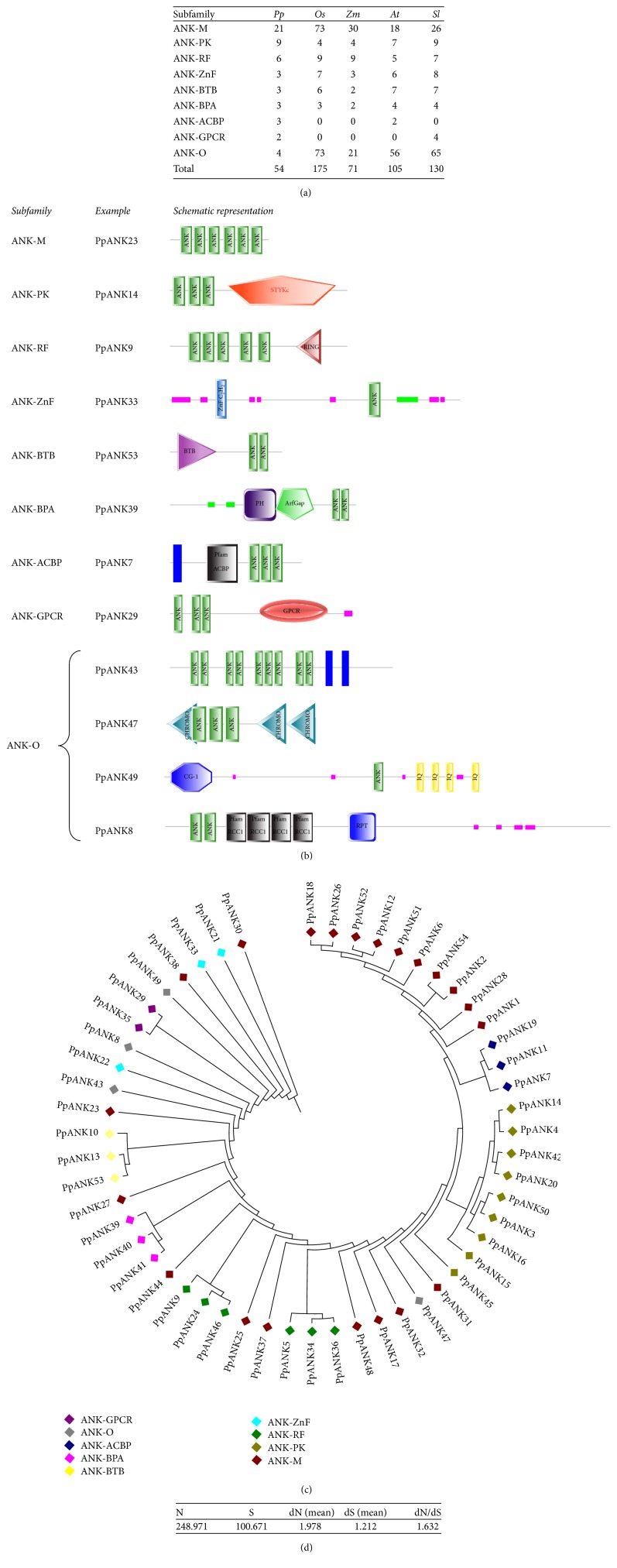
(a) Number of ANK proteins in each subfamily in* P. patens*, rice, maize,* Arabidopsis,* and tomato denoted as* Pp, Os, Zm, At,* and* Sl*, respectively. (b) Schematic representation of the structure of representative PpANK proteins from each subfamily. The figures shown here are not drawn to scale. (c) Evolutionary tree constructed from the full-length protein sequences of PpANK proteins. Different colors correspond to different subfamilies which are described in the right side of the tree. In most cases, the members of the same subfamily were clustered together. (d) Synonymous divergence (dS) and nonsynonymous divergence (dN) ratios of the ANK genes in* P. patens*.

The consensus ankyrin repeat sequence in* P. patens*, [ND]AxDKDGRT[PA]LHLAAxxGHxE[VA]-V[EK]LLLD[AH]GA[DN][VP], was generated by MEME webserver (http://meme.sdsc.edu/meme/intro.html) and visualized by Weblogo [26] as shown in Figure 2(a). The consensus ANK sequence in* P. patens* had a length of 33 amino acids and was conserved at the residues that are needed to retain the stacked L-shaped structure for protein-protein interaction, as mentioned by Mosavi and colleagues [27].

Based on their domain compositions, the predicted PpANK proteins were classified into nine subfamilies (Figure 2(a)). We have observed that a significant number of the PpANK proteins (21) had no other recognizable domain apart from the conserved ankyrin repeat and were classified as ANK-M. Proteins containing other known functional domains apart from the ANK domains were classified into the following subfamilies. Six proteins containing the RING finger domains were grouped as ANK-RF; three proteins containing the zinc-finger domain were designated as ANK-ZnF. BAR, PH and ArfGap domain containing proteins were grouped as ANK-BPA (3 members). The ANK-BTB subfamily (3 members) had broad-complex, tramtrack, and bric-a-brac domains. Nine of the PpANK proteins having either serine/threonine or tyrosine kinase domain were classified as ANK-PK. Three proteins having the Acetyl-CoA binding domain were classified as ACBP. Two proteins having the GPCR-chapero-1 domain were classified as ANK-GPCR. This specific subfamily containing the GPCR domain has only been reported to be found in tomato and has not been reported in model plant species like* Arabidopsis* and rice [16] (Figure 2(a)). The rest of the PpANK proteins that contained other domains including CHROMO, IQ, TM, and RCC1 were grouped as ANK-O. The structure of representative proteins from each subfamily is shown in Figure 2(b). There were no ANK proteins having the TPR domains (ANK-TPR) in* P. patens*, even though ANK proteins having these two domains are present in both* Arabidopsis* and rice [14, 15].

Next, we constructed a phylogenetic tree to compare between the members of different subfamilies of PpANKs. The tree file was generated from the Hidden Markov Model (HMM) based multiple sequence alignments of the sequences done by SATCHMO-JS and visualized by the Molecular Evolutionary Genetics Analysis (MEGA) software version 4.1 [23]. Interestingly, in most of the cases, members of the same subfamily were clustered together in the phylogenetic tree (Figure 2(c)).

We also analyzed the synonymous and nonsynonymous substitution patterns of the coding sequences of the genes encoding the ANK proteins in* P. patens*. The corresponding nucleotide sequences of the PpANK proteins were obtained from NCBI. Then we aligned the sequences using MEGA 4.1 and obtained the synonymous divergence (dS) and nonsynonymous divergence (dN) ratios. The ratio suggested positive selection for the genes of ANK superfamily of* P. patens* (Figure 2(d)). The codon based* Z* test indicated positive selection (data not shown) for most of the pairwise comparisons of the ANK genes. This further explains the fact that the ANK repeat encoding genes have acquired significant functional diversity by extensive domain shuffling or emerged multiple times independently, as a result of convergent evolution or parallel evolution or both [21].

**Figure 3:**
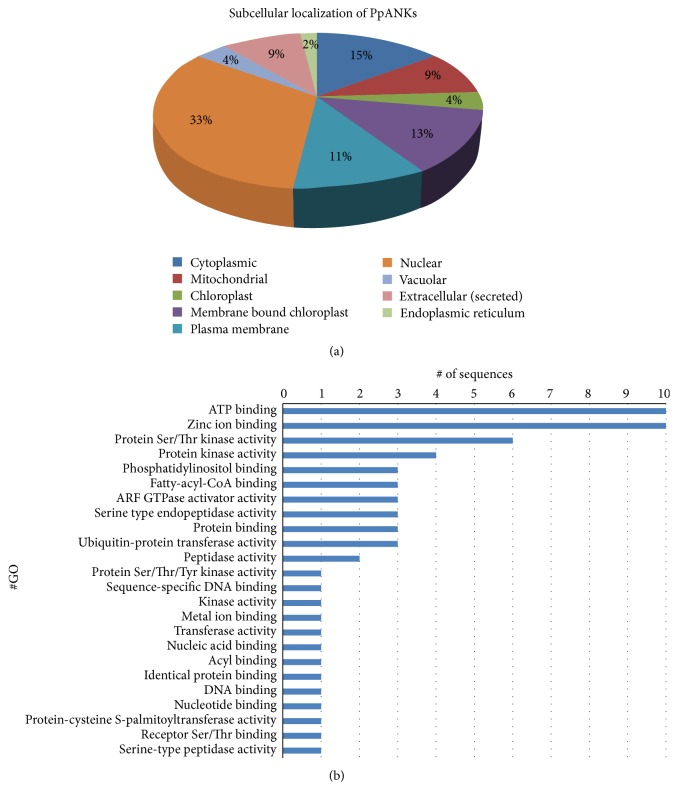
(a) Percentage distribution of PpANKs in different locations of the cells as predicted by ProtComp version 9.0. (b) Distribution of molecular function of the PpANK proteins as obtained from Blast2Go [13].

In order to elucidate the function of a protein within a living cell, predicting the location where it resides in the cell is essential. In this study we have used ProtComp version 9.0 for predicting the subcellular localization of the PpANK proteins. The output revealed that the proteins are dispersed throughout the cells (Figure 3(a)). A large percentage (33%) of the PpANKs are located in the nucleus. Detailed information on the localization of each protein can be found in Supplementary Table  1 in Supplementary Material available online at http://dx.doi.org/10.1155/2016/9156735. We also tried to analyze if there is any relationship between the subfamilies of PpANKs with their respective subcellular localization. Interestingly, we have found that all the members of the ANK-BPA subfamily had similar localization pattern, that is, in the extracellular region (Additional File 1, Supplementary Table  1). For all the other subfamilies, we did not see any distinct pattern in their localization.

The PpANK sequences were also compared with the proteins in of NCBI nonredundant protein database which showed their homology with ANK proteins from diverse species ranging from bacteria to green algae to plants (Additional File 1, Supplementary Table  2). Not surprisingly, in many of the cases, the proteins having significant similarity with the corresponding PpANKs have functions either as protein binders or as kinases (Figure 3(b)). This further clarifies the fact that ANK proteins play significant role in protein-protein interaction and cellular signaling pathways.

## 4. Conclusion

This study mainly focused on the sequences ANK proteins: their classification and phylogenetic analysis by using the first release of the moss genome annotation. We are aware that newer versions of the moss genome annotation are already available in Phytozome. As such the results shown here do not provide a complete overview of the whole repertoire of* P. patens* ankyrin proteins. Moreover, experimental verification and wet-lab functional studies of the genes encoding these proteins are necessary to come to any definite conclusion about their biological function. Nevertheless this may serve as a useful reference for more detailed functional analyses as well as for the selection of appropriate candidate genes for further studies and genetic manipulation of* P. patens* ankyrin proteins.

## Supplementary Material

Tables containing details of subcellular localization of each of the ANK proteins listed in this paper as well as their best similar homolog in other species are presented in Additional File 1.

## Figures and Tables

**Table 1:** List of ANK proteins identified in *P. patens*.

NCBI accession number	Our nomenclature	# of repeats	Length (A.A.s)	MW (KD)	PI
XP_001770032	PpANK1	3	176	18.54	4.02
XP_001770249	PpANK2	4	211	22.88	9.52
XP_001771297	PpANK3	2	425	47.57	8.63
XP_001774650	PpANK4	1	418	47.15	7.01
XP_001775781	PpANK5	4	364	40.21	8.24
XP_001756372	PpANK6	3	144	15.3	4.43
XP_001756378	PpANK7	3	376	41.11	4.48
XP_001777467	PpANK8	2	1088	116.86	8.18
XP_001779325	PpANK9	5	425	45.81	6.71
XP_001757508	PpANK10	2	463	50.67	7.19
XP_001782286	PpANK11	3	350	38.41	4.15
XP_001782585	PpANK12	3	121	12.8	6.29
XP_001759240	PpANK13	2	440	48.17	6.19
XP_001783731	PpANK14	3	421	47.49	7.01
XP_001784155	PpANK15	3	442	49.87	7.19
XP_001765240	PpANK16	3	419	47.13	7.75
XP_001765521	PpANK17	3	167	18.4	8.75
XP_001753203	PpANK18	1	82	8.73	4.67
XP_001766044	PpANK19	3	342	37.48	4.19
XP_001767452	PpANK20	2	412	46.92	8.12
XP_001771908	PpANK21	2	438	48.19	8.08
XP_001755743	PpANK22	1	291	31.45	8.6
XP_001784959	PpANK23	6	247	27.98	6.27
XP_001762570	PpANK24	5	437	47.16	7.64
XP_001764006	PpANK25	2	337	37.55	4.44
XP_001753287	PpANK26	3	343	36.03	4.09
XP_001774971	PpANK27	3	378	41.73	5.74
XP_001779754	PpANK28	4	234	25.26	8.85
XP_001759321	PpANK29	3	608	68.4	6.69
XP_001760427	PpANK30	2	377	41.77	6.23
XP_001765151	PpANK31	4	625	69.45	5.03
XP_001765275	PpANK32	4	351	37.69	6.85
XP_001767776	PpANK33	1	752	82.78	9.39
XP_001771059	PpANK34	4	451	48.45	5.64
XP_001780358	PpANK35	3	634	71.25	8.42
XP_001763413	PpANK36	4	473	50.65	5.08
XP_001773791	PpANK37	3	271	30.45	4.58
XP_001773863	PpANK38	2	107	11.37	3.88
XP_001774458	PpANK39	2	731	82.73	7.11
XP_001779036	PpANK40	2	721	82.01	7.12
XP_001759158	PpANK41	2	752	84.75	8.64
XP_001784362	PpANK42	3	414	47.27	6.51
XP_001765211	PpANK43	9	795	90.39	7.16
XP_001770763	PpANK44	6	1060	114.9	8.03
XP_001765353	PpANK45	3	612	67.04	8.35
XP_001754260	PpANK46	5	402	43.17	7.72
XP_001771412	PpANK47	3	285	32.42	4.04
XP_001755344	PpANK48	2	74	7.65	4.34
XP_001775223	PpANK49	1	910	101.79	7.62
XP_001758257	PpANK50	3	380	42.3	8.66
XP_001758416	PpANK51	3	86	9.13	7.16
XP_001782553	PpANK52	3	111	11.72	6.67
XP_001784176	PpANK53	2	367	40.47	7.52
XP_001760375	PpANK54	3	130	14.03	10.41
