# Supplementary material for: Analyses of Physcomitrella patens Ankyrin Repeat Proteins by Computational Approach
Source: Mol Biol Int. 2016 Jun 27;2016:9156735. doi: 10.1155/2016/9156735 (PMC4939350; doi:10.1155/2016/9156735)
Supplement: Supplementary file 1 — Tables containing details of subcellular localization of each of the ANK proteins listed in this paper as well as their best similar homolog in other species are presented in Additional File 1. [file 9156735.f1.pdf]

Supplementary Table 1: Sub-cellular localization of the PpANK proteins predicted by ProtComp Version 9.0.

PpANK protein	Subfamily	Subcellular localization
**PpANK1**	ANK-O	Cytoplasmic
**PpANK2**	ANK-M	Mitochondrial
**PpANK3**	ANK-PK	Mitochondrial
**PpANK4**	ANK-PK	Membrane bound Chloroplast
**PpANK5**	ANK-RF	Membrane bound Chloroplast
**PpANK6**	ANK-O	Membrane bound Chloroplast
**PpANK7**	ANK-O	Plasma membrane
**PpANK8**	ANK-O	nuclear
**PpANK9**	ANK-RF	Cytoplasmic
**PpANK10**	ANK-BTB	nuclear
**PpANK11**	ANK-O	Plasma membrane
**PpANK12**	ANK-M	Chloroplast
**PpANK13**	ANK-BTB	Plasma membrane
**PpANK14**	ANK-PK	Membrane bound Chloroplast
**PpANK15**	ANK-PK	Plasma membrane
**PpANK16**	ANK-PK	Cytoplasmic
**PpANK17**	ANK-M	nuclear
**PpANK18**	ANK-M	Vacuolar
**PpANK19**	ANK-O	Extracellular (Secreted)
**PpANK20**	ANK-PK	Cytoplasmic
**PpANK21**	ANK-ZnF	Plasma membrane
**PpANK22**	ANK-ZnF	Mitochondrial
**PpANK23**	ANK-M	nuclear
**PpANK24**	ANK-RF	nuclear
**PpANK25**	ANK-M	Cytoplasmic
**PpANK26**	ANK-M	nuclear
**PpANK27**	ANK-M	nuclear
**PpANK28**	ANK-M	Mitochondrial
**PpANK29**	ANK-O	nuclear
**PpANK30**	ANK-M	Membrane bound Chloroplast
**PpANK31**	ANK-M	nuclear
**PpANK32**	ANK-M	nuclear
**PpANK33**	ANK-ZnF	nuclear
**PpANK34**	ANK-RF	nuclear
**PpANK35**	ANK-M	nuclear

**PpANK36**	ANK-RF	nuclear
**PpANK37**	ANK-M	Vacuolar
**PpANK38**	ANK-M	Mitochondrial
**PpANK39**	ANK-BPA	Extracellular (Secreted)
**PpANK40**	ANK-BPA	Extracellular (Secreted)
**PpANK41**	ANK-BPA	Extracellular (Secreted)
**PpANK42**	ANK-PK	Cytoplasmic
**PpANK43**	ANK-M	Plasma membrane
**PpANK44**	ANK-M	nuclear
**PpANK45**	ANK-PK	nuclear
**PpANK46**	ANK-RF	Cytoplasmic
**PpANK47**	ANK-PK	Membrane bound Chloroplast
**PpANK48**	ANK-M	Chloroplast
**PpANK49**	ANK-O	nuclear
**PpANK50**	ANK-PK	Membrane bound Chloroplast
**PpANK51**	ANK-M	Endoplasmic reticulum
**PpANK52**	ANK-M	Extracellular (Secreted)
**PpANK53**	ANK-BTB	Cytoplasmic
**PpANK54**	ANK-M	Nuclear

Supplementary Table 2: Blast-P of the PpANK proteins showing the species with best similar homolog, their accession numbers and the E-values .

Our Nomenclature	Species with best match	Type of the species with best match	Acsession Number	E-value
**PpANK1**	*Theobroma cacao*	dicot plant	EOY07972	5.00E-49
**PpANK2**	*Theobroma cacao*	dicot plant	EOY03438	4.00E-33
**PpANK3**	*Theobroma cacao*	dicot plant	EOX95980	9.00E-117
**PpANK4**	*Populus trichocarpa*	dicot plant	XP_002312262	2.00E-127
**PpANK5**	*Arabidopsis thaliana*	dicot plant	NP_567428	7.00E-95
**PpANK6**	*Zea mays*	monocot plant	NP_001141239	1.00E-40
**PpANK7**	*Agave americana*	monocot plant	AAT81164	1.00E-62
**PpANK8**	*Populus trichocarpa*	monocot plant	XP_002319149	0.00E+00
**PpANK9**	*Theobroma cacao*	dicot plant	EOY23127	3.00E-174
**PpANK10**	*Theobroma cacao*	dicot plant	EOY09068	1.00E-117
**PpANK11**	*Populus trichocarpa*	dicot plant	XP_002321391	2.00E-82
**PpANK12**	*Trichomonas vaginalis*	protozoan , microorganism	*XP_001312218*	1.00E-21
**PpANK13**	*Theobroma cacao*	dicot plant	EOY09068	4.00E-110
**PpANK14**	*Ricinus communis*	dicot plant	*XP_002522482*	2.00E-128
**PpANK15**	*Theobroma cacao*	dicot plant	EOX92972	1.00E-129
**PpANK16**	*Theobroma cacao*	dicot plant	EOX95980	2.00E-121
**PpANK17**	*Zea mays*	monocot plant	NP_001140799	1.00E-67
**PpANK18**	*Triticum urartu*	monocot plant	EMS53267	3.00E-38
**PpANK19**	*Agave americana*	monocot plant	AAT81164	1.00E-72
**PpANK20**	*Zea mays*	monocot plant	DAA63750	8.00E-144
**PpANK21**	*Theobroma cacao*	dicot plant	EOY31240	8.00E-149
**PpANK22**	*Aegilops tauschii*	monocot plant	EMT07606	2.00E-77
**PpANK23**	*Wolbachia pipientis*	bacteria	AEX55224	3.00E-25
**PpANK24**	*Theobroma cacao*	dicot plant	EOY31606	0.00E+00
**PpANK25**	*Theobroma cacao*	dicot plant	EOY15770	1.00E-169
**PpANK26**	*Zea mays*	monocot plant	NP_001183774	5.00E-155
**PpANK27**	*Arabidopsis thaliana*	dicot plant	AAA32812	3.00E-32
**PpANK28**	*Ricinus communis*	dicot plant	XP_002519880	1.00E-54
**PpANK29**	*Theobroma cacao*	dicot plant	EOY10889	0.00E+00
**PpANK30**	*Leptospira borgpetersenii*	bacteria	WP_002755186	1.30E+00

**PpANK31**	*Populus trichocarpa*	dicot plant	XP_006372521	6.00E-41
**PpANK32**	uncultured *Acidilobus* sp. JCHS	Archaea	WP_023429631	3.00E-23
**PpANK33**	*Populus trichocarpa*	dicot plant	XP_006386575	4e-129
**PpANK34**	*Theobroma cacao*	dicot plant	EOY06059	5.00E-117
**PpANK35**	*Ricinus communis*	dicot plant	XP_002521987	0.00E+00
**PpANK36**	*Theobroma cacao*	dicot plant	EOY06059	3.00E-115
**PpANK37**	*Chlamydomonas reinhardtii*	green algae	XP_001699643	4.00E-19
**PpANK38**	*Perkinsus marinus* ATCC 50983	protist	XP_002774879	1.00E-05
**PpANK39**	*Populus trichocarpa*	dicot plant	XP_002322301	0.00E+00
**PpANK40**	*Populus trichocarpa*	dicot plant	XP_002322301	0.00E+00
**PpANK41**	*Populus trichocarpa*	dicot plant	XP_002303749	0.00E+00
**PpANK42**	*Theobroma cacao*	dicot plant	EOY01835	5.00E-147
**PpANK43**	*Talaromyces stipitatus* ATCC 10500	fungus	XP_002340125	4.00E-34
**PpANK44**	*Volvox carteri*	green algae	XP_002957021	1.00E-29
**PpANK45**	*Medicago truncatula*	dicot plant	XP_003629944	2.00E-24
**PpANK46**	*Theobroma cacao*	dicot plant	EOY23127	0.00E+00
**PpANK47**	*Populus trichocarpa*	dicot plant	XP_002313685	3.00E-98
**PpANK48**	*Trichomonas vaginalis* G3	protozoan , microorganism	XP_001316774	3.00E-12
**PpANK49**	*Arabidopsis lyrata*	dicot plant	XP_002871369	0.00E+00
**PpANK50**	*Theobroma cacao*	dicot plant	EOX95980	5.00E-127
**PpANK51**	*Persephonella marina* EX-H1	bacteria	YP_002730164	6.00E-20
**PpANK52**	*Trichomonas vaginalis* G3	protozoan , microorganism	XP_001301368	3.00E-23
**PpANK53**	*Theobroma cacao*	dicot plant	EOY09068	2.00E-119
**PpANK54**	*Theobroma cacao*	dicot plant	EOY03438	7.00E-26
